# IFT140^+^/K14^+^ cells function as stem/progenitor cells in salivary glands

**DOI:** 10.1038/s41368-022-00200-5

**Published:** 2022-10-10

**Authors:** Xueming Zhang, Ji Zhou, Xinyu Wang, Jiangyu Geng, Yubei Chen, Yao Sun

**Affiliations:** 1 Department of Oral and Maxillofacial Surgery, Stomatological Hospital and Dental School of Tongji University, Shanghai Engineering Research Center of Tooth Restoration and Regeneration, No. 399, YanChang Middle Road, Shanghai, China; 2 Department of Implantology, Stomatological Hospital and Dental School of Tongji University, Shanghai Engineering Research Center of Tooth Restoration and Regeneration, No. 399, YanChang Middle Road, Shanghai, China

**Keywords:** Regeneration, Protein transport

## Abstract

Stem/progenitor cells are important for salivary gland development, homeostasis maintenance, and regeneration following injury. Keratin-14^+^ (K14^+^) cells have been recognized as bona fide salivary gland stem/progenitor cells. However, K14 is also expressed in terminally differentiated myoepithelial cells; therefore, more accurate molecular markers for identifying salivary stem/progenitor cells are required. The intraflagellar transport (IFT) protein IFT140 is a core component of the IFT system that functions in signaling transduction through the primary cilia. It is reportedly expressed in mesenchymal stem cells and plays a role in bone formation. In this study, we demonstrated that IFT140 was intensively expressed in K14^+^ stem/progenitor cells during the developmental period and early regeneration stage following ligation-induced injuries in murine submandibular glands. In addition, we demonstrated that IFT140^+^/ K14^+^ could self-renew and differentiate into granular duct cells at the developmental stage in vivo. The conditional deletion of *Ift140* from K14^+^ cells caused abnormal epithelial structure and function during salivary gland development and inhibited regeneration. IFT140 partly coordinated the function of K14^+^ stem/progenitor cells by modulating ciliary membrane trafficking. Our investigation identified a combined marker, IFT140^+^/K14^+^, for salivary gland stem/progenitor cells and elucidated the essential role of IFT140 and cilia in regulating salivary stem/progenitor cell differentiation and gland regeneration.

## Introduction

Salivary glands secrete saliva, which is essential for maintaining oral microenvironments and health. However, salivary glands are prone to be damaged under several conditions, including radiation therapies targeting the head and neck region and autoimmune diseases such as Sjögren’s syndrome.^1^ Regenerative approaches based on activating tissue-resident stem/progenitor cells could potentially restore gland structure and function.^2–6^ Keratin-14^+^ (K14^+^) cells have gained increasing attention given that they are stem/progenitor cells that are actively involved in salivary gland development, homeostasis maintenance, and regeneration following injury.^4,7–9^ Nevertheless, K14^+^ cells include diverse subpopulations of salivary stem/progenitor cells, such as K14^+^/c-Kit^+^ or K14^+^/K5^+^ lineages, playing different roles during development or homeostasis.^10,11^ In addition, K14 is also expressed in the terminally differentiated myoepithelial cells.^3,7^ Therefore, it is important to define more specific and indicative markers for salivary gland stem/progenitor cells.

The primary cilium is a sensor organelle involved in stem cell differentiation and renewal by regulating multiple signaling pathways.^12^ The intraflagellar transport (IFT) system is critical for building the cilium and shuttling signaling receptors through the primary cilia.^13–15^ We had reported an IFT complex core component IFT140 that was expressed in the mesenchymal stem/progenitor cells and played an important role in cell differentiation during tooth and bone development.^16–19^ In humans, mutations in *Ift140* lead to ciliopathies that exhibit multi-system maldevelopment and disfunction.^20–26^ Although *Ift140* mutation has not been related to salivary gland dysfunction, some other IFT protein mutations were reported to cause abnormalities in exocrine glands such as pancreas.^27^ We recently evaluated the gene expression of several member proteins of the IFT system during salivary gland regeneration and found that *Ift140* expression level was leadingly increased at the early stage of regeneration, accompanied by an increase in the number of proliferating K14^+^ cells. This led us to assess the relationship between the function of IFT140 and the activation of K14^+^ stem/progenitor cells, and to analyze whether IFT140 plays an active role in the development and regeneration of salivary glands.

In this study, we demonstrate that IFT140 was intensively expressed in K14^+^ stem/progenitor cells during the salivary gland development period and the early regeneration stage following ligation-induced injury. A genetic lineage tracing study indicated that IFT140^+^ cells co-expressed with K14 were capable of self-renewal and differentiating into granular duct cells. The selective deletion of *Ift140* from K14^+^ cells caused abnormal salivary development and inhibited regeneration. Moreover, we found that IFT140 could regulate the function of K14^+^ stem/progenitor cells by modulating ciliary membrane trafficking. As such, our study identifies IFT140^+^/K14^+^ as an indictive marker for salivary gland stem/progenitor cells during gland development and regeneration in vivo.

## Results

### IFT140 participates in salivary gland regeneration following ligation-induced injury

**Fig. 1:**
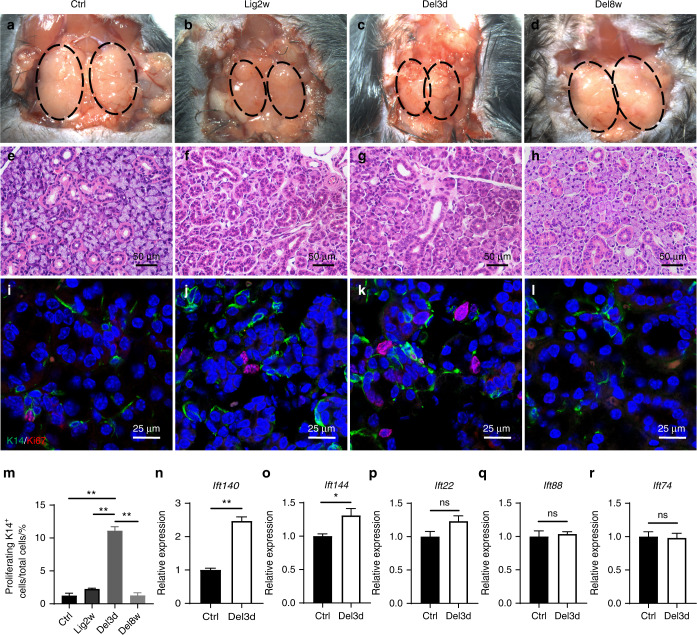
IFT140 participates in salivary gland regeneration following ligation-induced injury. **a**–**d** Main ducts of SMGs of adult wild-type mice are ligated for 2 weeks and deligated to observe the regenerating process. The gross feature of the SMGs at different time points are presented. The dotted lines outline the glands. Ctrl normal unligated glands; Lig2w ligated for 2 weeks (injury period); Del3d 3rd day after de-ligation (the early stage of regeneration); Del8w 8 weeks after de-ligation (the late stage of regeneration). **e**–**h** H&E staining shows the atrophic and fibrotic change of the injured glands in 2 weeks’ ligation and almost a full restoration within 8 weeks following de-ligation. **i**–**l** K14^+^ cells were lmmunolabelled and Ki67 was used to label the proliferating cells. **m** Quantitative analysis of the ratio of K4^+^/Ki67^+^ cells to total cells at different time points. One-way ANOVA, *n* = 6, **P < 0.01. **n**–**r** Multiple key molecules in IFT system were detected by real-time PCR. Unpaired Student’s *t* test or Welch’s *t*-test, *n* = 6, *P < 0.05, **P < 0.01. Scale bar: 50 or 25 μm as indicated

To investigate whether IFT proteins are involved in the salivary gland regeneration process, we used a classic duct ligation-induced injury model in female adult mice. The 2-week ligation process decreased gland volume (Fig. 1a, b). Histological analysis of the SMGs 2 weeks post ligation revealed severe atrophy of the acini and a luminal enlargement of GDs due to the loss of secretory granules (Fig. 1e, f). No improvements in the gross or histological features of the glands were observed on day 3 post de-ligation (Fig. 1c, g), whereas glands appeared fully restored within 8 weeks (Fig. 1d, h). Immunofluorescent staining of SMG sections revealed a rapid expansion in the K14^+^ cell population at day 3 post de-ligation, resulting in a significant increase in the proportion of proliferating K14^+^ cells (Fig. 1i–l, quantified in m).

Next, we evaluated the expression level of several member molecules that compose IFT-A (*Ift140, Ift144*) or IFT-B (*Ift22, Ift88, Ift74*) complex at the early stage of regeneration (day 3 post de-ligation). Real-time PCR results showed that *Ift140* expression was leadingly elevated while the IFT-B complex members remained at the base level (Fig. 1n–r). These results suggested that IFT140 might take an active part in salivary gland regeneration at the early stage and relate to the activation of K14^+^ stem/progenitor cells.

### IFT140 expression in K14^+^ stem/progenitor cells coincide with their differentiation in salivary gland development

**Fig. 2:**
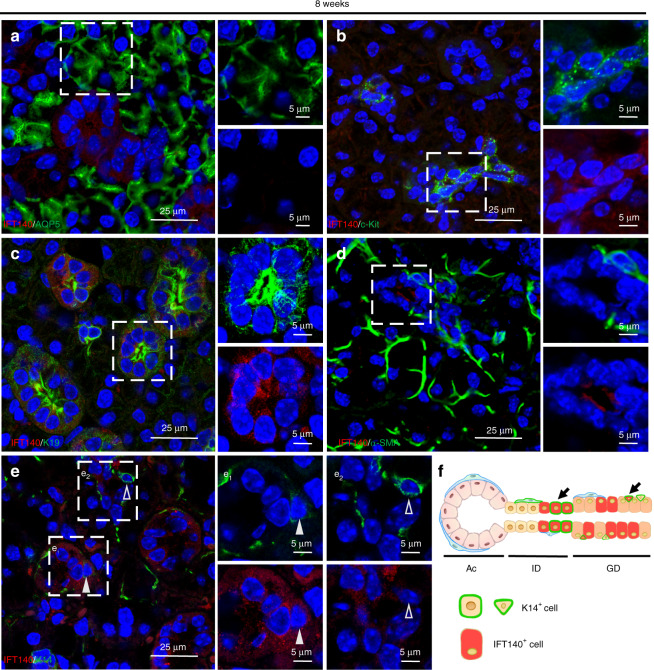
Distribution of IFT140^+^ cells in murine submandibular glands (SMGs). **a**–**e** Co-immunostaining of IFT140 and markers for different epithelial cell types of SMGs in 8-week-old female mice (AQP5 marks acinar cells; c-Kit marks intercalated duct cells; K19 marks granular duct cells; α-SMA marks myoepithelial cells; K14 marks K14^+^ cells). IFT140 was expressed in some K14^+^ cells (**e**1, solid arrowheads) but not in others (**e**2, hollowed arrowheads). The boxed areas were magnified and presented on the right panels. The green and red channels were separately presented for a clearer view. **f** The schematic diagram of the distribution of IFT140^+^ cells. Scale bar: 25 or 5 μm as indicated

To systematically investigate the spatial expression pattern of IFT140 in SMG epithelium, we used double immunolabeling with antibodies against IFT140 and the respective markers for acini (anti-AQP5), intercalated ducts (ID, anti-c-Kit), granular ducts (GD, anti-K19), myoepithelial cells (MECs, anti-α-SMA) and K14^+^ cells in 8-week-old female C57BL/6 mice.^7^ IFT140 was moderately expressed in a portion of ID and GD cells but was absent in acinar cells and MECs (Fig. 2a–d, diagrammed in f). Notably, IFT140 expression was only detected in a few K14^+^ cells (Fig. 2e).

**Fig. 3:**
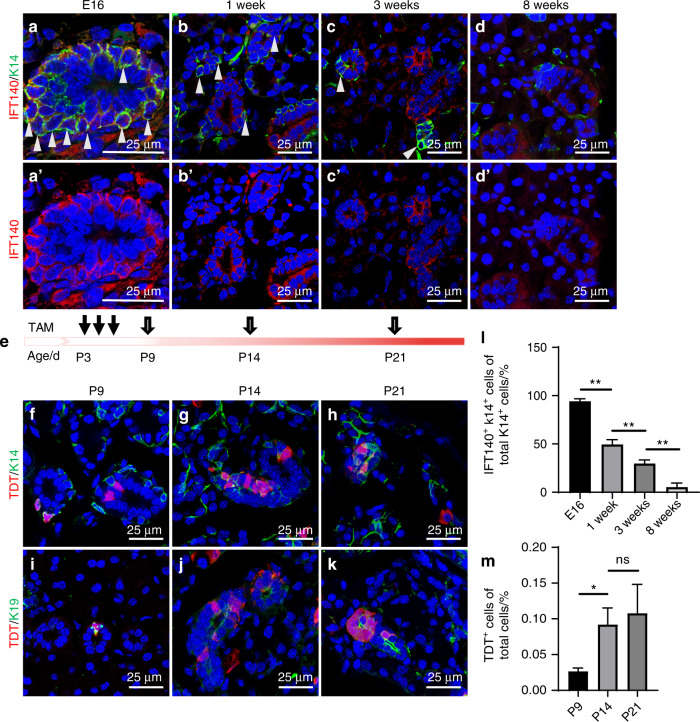
IFT140 expression pattern in K14^+^ stem/progenitor cells coincides with their differentiation in SMG development. **a**–**d** Co-immunostaining of IFT140 and K14 for SMG tissues from different stages of development. Arrowheads indicate IFT140^+^/K14^+^ cells. The ratio of IFT140^+^/K14^+^ cells to total K14^+^ cells is quantified in **l**. One-way ANOVA, *n* = 6. **P < 0.01. **e** The strategy used for genetic labeling of IFT140-expressing cells with tdTomato (TdT) in newborn mice. Tamoxifen (TAM) was administrated at postnatal day (P) 3, P5, P7, and glands were harvested at P9, P14, and P21. **f**–**k** Co-immunostaining of TdT-labeled cells with K14 or K19 (marker for granular duct cells). The ratio of TdT^+^ cells to total cells is quantified in **m**. One-way ANOVA, *n* = 6. ns no significance; **P < 0.01. E embryo; P postnatal. Scale bar: 25 μm

Based on these results, we investigated whether IFT140 expression was associated with specific developmental cues by examining its expression in K14^+^ cells in SMGs from multiple stages of mouse development, from the embryo (E) through maturity stages. Quantitative image analysis showed that IFT140 was highly expressed in 95.1% of the K14^+^ cells at E16, the specific stage at which SMGs undergo terminal differentiation into secretory cell types.^3^ However, the ratio of IFT140-expressing K14^+^ cells to total K14^+^ cells decreased over time, declining to 4.5% at 8 weeks (Fig. 3a–d, quantified in l). This reduction could be due to the increasing quantity of the IFT140^−^/K14^+^ MECs long with gland maturation. In light of our finding that IFT140 was expressed in a larger proportion of K14^+^ cells in the fast development period but less during maturation, we hypothesize that IFT140^+^/K14^+^ represents the tissue-resident stem/progenitor cells in salivary glands.

To test this hypothesis, we next used a genetic lineage tracing approach to analyze the fate of IFT140^+^ cells and their progeny during SMG development. To this end, we mated *Ift140-CreER* mice with *Rosa26*^*tdTomato*^ mice to generate an *Ift140-TdT* line. Tamoxifen (TAM) was injected on Postnatal day (P) 3, P5, P7, respectively, and SMGs were harvested at P9, P14, and P21 (Fig. 3e). The results demonstrated that the percentage of TdT-labeled cells increased over time (Fig. 3m). On P9, some of the K19^+^ GD cells or K14^+^ cells were labeled by TdT (Fig. 3f, i). On P14 and P21, an increased number of TdT-labeled K19^+^ cells were found in GDs in clusters over time (Fig. 3i–k). The TdT-labeled GD cells were just located adjacent to the K14^+^ cells, indicating clones of GD cells expanded from IFT140^+^/K14^+^ cells (Fig. 3f–h). These results coincided with the reported fate of K14^+^ stem/progenitor cells during postnatal development,^8^ and indicated that IFT140 participated in K14^+^ stem/progenitor cell differentiation during salivary gland development.

### *Ift140* deletion in K14^+^ cells lead to defects in salivary gland development

**Fig. 4:**
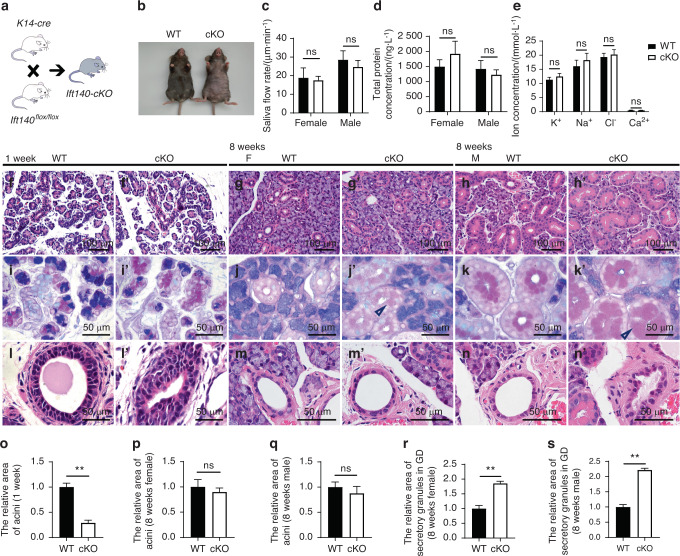
The phenotype of SMGs in developing and mature *Ift140-cKO* mice. **a** By mating *Ift140*^*flox/flox*^ mice with *K14-cre* mice, *Ift140* was selectively knocked out from K14^+^ cells. **b** The appearance of the adult female wild-type (WT) and *Ift140-cKO* (cKO) mice. **c**–**e** The saliva flow rate, concentration of total protein and ion of the whole saliva of adult WT and *Ift140-cKO* mice. Unpaired Student’s *t* test or Mann–Whitney *U* test was used, *n* = 6. ns no significance. **f**–**h** H&E staining of SMGs of different weeks of age and sex of WT and *Ift140-cKO* mice. F female; M male. **i**–**k** AB/PAS staining of SMGs. Acini are stained purple-blue. Hollow arrows indicate secretory granules (purple-red) in granular duct cells. **l**–**n** H&E staining shows the interlobular ducts. The relative area of acini (**o**–**q**) and area of secretory granules in granular ducts (**r**, **s**) were quantified by image analysis. Unpaired Student’s *t* test, *n* = 6. ns no significance; **P < 0.01. Scale bar: 50 or 100 μm as indicated

To further investigate whether and how IFT140 participates in salivary epithelial development, we selectively deleted *Ift140* from K14^+^ cells by mating *Ift140*^*fl/fl*^ mice with *K14-Cre* mice (Fig. 4a). These *Ift140* conditional knockout (*Ift140-cKO*) mice exhibited a sparse hair phenotype (Fig. 4b). There were no significance differences in the stimulated saliva flow rate or the concentration of total proteins and ions of the whole saliva between adult wild-type (WT) and *Ift140-cKO* mice (Fig. 4c–e). H&E staining of glands from 1-week-old *Ift140-cKO* mice revealed a smaller acini area than those of the WT mice, with subsequent quantification indicating that the acinar area in *Ift140-cKO* glands was 28.5% of that of WT glands (Fig. 4f, i, o). However, there was no significance difference in the acinar area between adult *Ift140-cKO* and WT mice in both sexes (Fig. 4g, h, p, q). Moreover, *Ift140-cKO* mice exhibited excessive accumulation of secretory granules in GDs (Fig. 4j, k). The area of secretory granules in adult female *Ift140-cKO* mice was 1.9-fold higher than that in WT mice, and it was 2.2 folds in males (Fig. 4r, s). H&E staining showed disorganized interlobular duct cells in *Ift140-cKO* mice (Fig. 4l–n). These results indicated that the selective deletion of *Ift140* from K14^+^ cells altered the histological structure in both acinar and ductal epithelium.

**Fig. 5:**
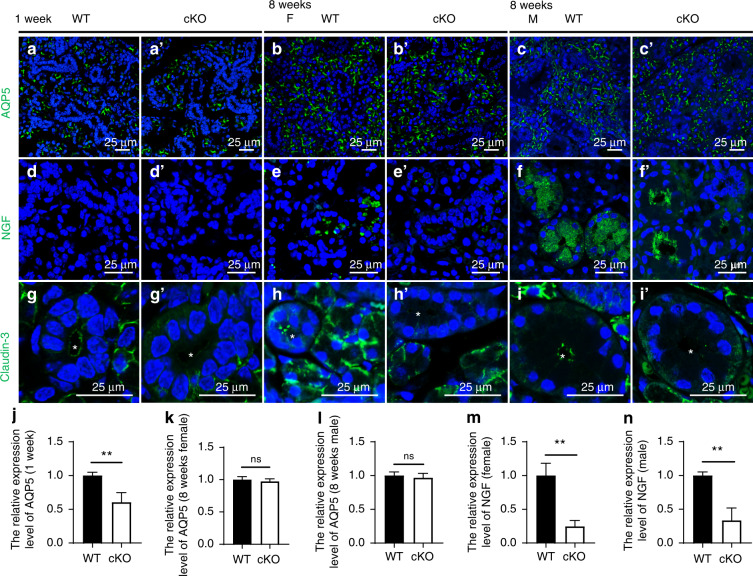
The altered expression of multiple proteins related to secretory function in *Ift140-cKO* mice. Immunostaining of aquaporin 5 (AQP5) in SMGs of 1-week-old (**a**), 8-week-old female (**b**) and 8-week-old male (**c**) mice. The relative expression level measured by fluorescence intensity in different samples as indicated is presented in **j**–**l**. Unpaired Student’s *t* test or Welch’s *t*-test, *n* = 6, ns no significance; **P < 0.01. **d**–**f** Immunostaining of nerve growth factor (NGF). Fluorescence intensity is quantified in **m**, **n**. Unpaired Student’s *t* test or Welch’s *t*-test, *n* = 6. **P < 0.01. **g**–**i** Immunostaining of Claudin-3. Asterisks indicate lumina of ducts. Scale bar: 25 μm

We next evaluated the function of the secretory cells by immunofluorescent staining. Aquaporin 5 (AQP5) mediates transcellular fluid transport in salivary glands.^28^ The results demonstrated that AQP5 expression decreased in acini of the 1-week-old cKO mice, although no difference was detectable between adult WT and I*ft140-cKO* mice (Fig. 5a–c, quantified in j–l). Nerve growth factor (NGF) is produced and stored in the secretory granules by GD cells beginning when mice are 2–3 weeks old.^29,30^ The staining and quantitative image analysis indicated that NGF contents significantly decreased in the GD cells of *Ift140-cKO* glands compared with WT (Fig. 5d–f, quantified in m, n), which suggested that granule contents were dysregulated in the absence of IFT140. Claudin-3 contributes to the structure of tight junctions, which maintain cell polarity and regulate paracellular fluid transport in salivary glands.^31,32^ Claudin-3 was expressed in the apicolateral membrane of the WT acinar or ductal cells but showed no expression in duct cells of either newborn or adult *Ift140-cKO* mice (Fig. 5g–i). Reminiscent of the disorganized duct cells in histological staining, these results indicated disorder in the polarity of duct cells and altered secretory function of GDs with the loss of *Ift140* in K14^+^ cells. These results revealed that *Ift140* deletion, which resulted in the dysregulation of K14^+^ stem/progenitor cell function, appeared to negatively impact the development of salivary epithelium.

### IFT140^+^/K14^+^ cells were required for epithelial regeneration

**Fig. 6:**
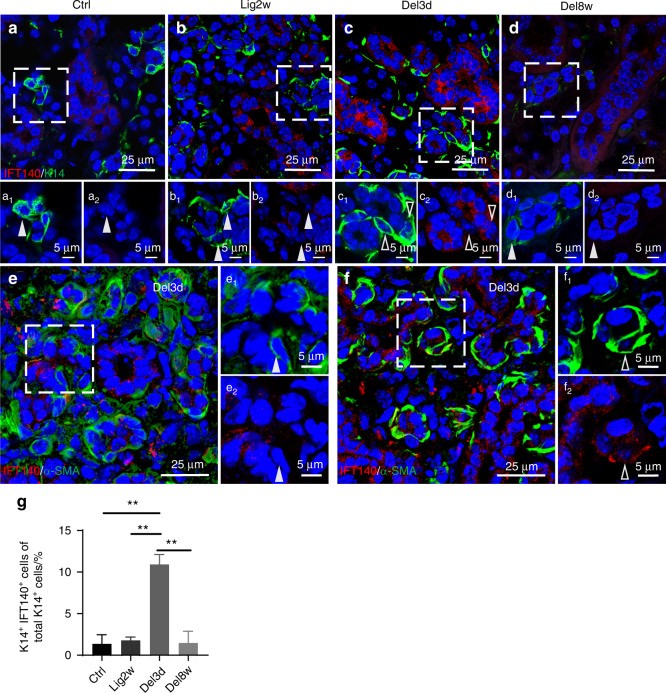
IFT140^+^/K14^+^ progenitor cells are activated at the early stage of gland regeneration. **a**–**d** Co-immunostaining of IFT140 with K14 in SMGs at representative time points in the gland injury model. Ctrl normal unligated glands; Lig2w ligated for 2 weeks; Del3d 3rd day after de-ligation; Del8w 8 weeks after de-ligation. **e**, **f** Co-immunostaining of IFT140 with α-SMA in SMGs on the 3rd day after de-ligation. The boxed areas are magnified, and the green and red channels are presented separately for a clearer view. Hollowed arrowheads indicate IFT140^+^ cells, and solid arrowheads indicate IFT140^−^ cells. **g** The ratio of IFT140^+^/K14^+^ cells to total K14^+^ cells was quantified. One-way ANOVA, *n* = 6. **P < 0.01. Scale bars are indicated in the figures

We next investigated whether IFT140 could affect the regeneration process of salivary gland through K14^+^ stem/progenitor cells using the ligation-induced salivary gland injury model. Immunostaining and quantitative analysis showed that the number of K14^+^ cells co-expressing IFT140 was significantly increased at day 3 post-de-ligation, the early stage of regeneration (Fig. 6a–d, quantified in g). Since terminally differentiated MECs also express K14, we further investigated whether MECs expressed IFT140 during regeneration. Interestingly, although MECs were IFT140 negative in most areas of the glands at day 3 post de-ligation (Fig. 6e), we found that IFT140 was highly expressed in MECs in regions with severe atrophy (Fig. 6f). Since MECs could revert into a progenitor-like state in response to a severe damage,^7^ it was reasonable to attribute their IFT140 expression to the acquisition of multipotency.

**Fig. 7:**
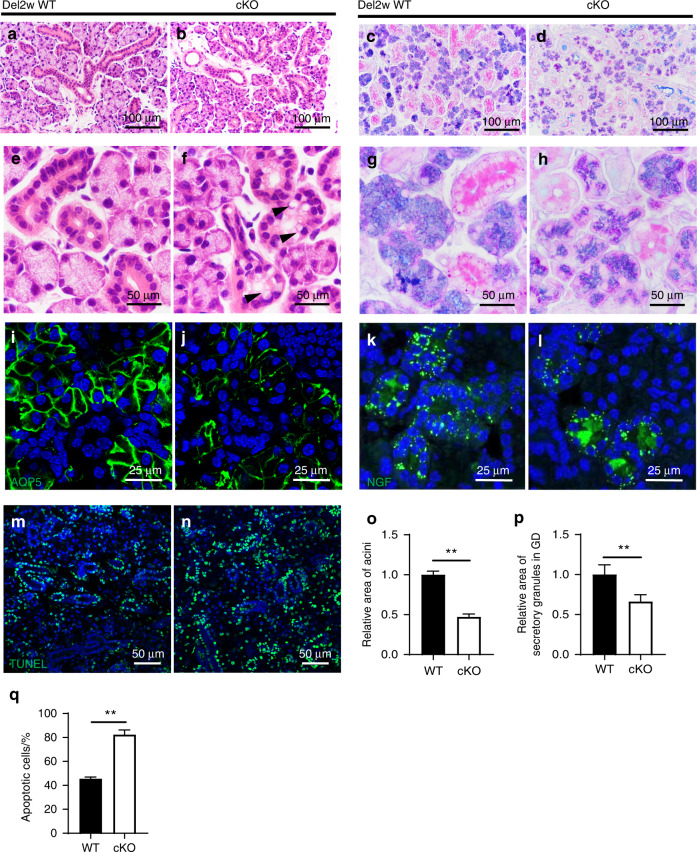
*Ift140* deletion in K14^+^ cells affects the salivary gland regenerating process. Comparisons were made between wild-type and *Ift140-cKO* mice at 2 weeks post de-ligation (Del2w), a midway point in regeneration. **a**, **b**, **e**, **f** H&E staining presented at both low and high-power fields. Arrowheads indicate cytoplasmic vacuolation. **c**, **d**, **g**, **h** AB/PAS staining. The relative area of acini and the secretory granules in GD were quantified in **o**, **p**. Unpaired Student’s *t* test, *n* = 6, **P < 0.01. **i**–**l** Immunostaining of aquaporin 5 (AQP5) and nerve growth factor (NGF). **m**, **n** TUNEL assay. Apoptotic cells are stained green. The ratio of apoptotic cells to total cells was quantified in **q**. Welch’s *t*-test, *n* = 6, **P < 0.01. Scale bars are indicated in the figures

To examine whether *Ift140* deletion in K14^+^ cells could also affect the salivary gland regeneration process, we compared the phenotypes of regenerating glands at 2 weeks post de-ligation (a midway point in regeneration) between WT and *Ift140-cKO* mice in the ligature-induced injury model. The results showed that morphological restoration was slower for *Ift140-cKO* glands at 2 weeks after de-ligation than for WT glands. Specifically, the *Ift140-cKO* glands decreased secretory granules in acinar and GD cells and wider interlobular spaces than those of WT glands (Fig. 7a–h). The average area of acini and secretory granules in GD cells of *Ift140-cKO* glands was 45.8% and 66.2% of those of WT glands, respectively (Fig. 7o, p). Cytoplasmic vacuolization was consistently observed in the ductal cells of *Ift140-cKO* glands (Fig. 7f). In addition, AQP5 expression significantly decreased in *Ift140-cKO* glands relative to WT (Fig. 7i, j), and NGF was unevenly distributed throughout ductal cells (i.e., clusters of ductal cells with high NGF levels were scattered stochastically in GDs) (Fig. 7k, l). TUNEL assays showed that the number of cells with DNA damage was significantly higher in *Ift140-cKO* glands than in WT glands, suggesting an inhibited recovery of gland in *Ift140-cKO* mice (Fig. 7m, n, quantified in q). These results indicated that IFT140 function was related to the activation of K14^+^ stem/progenitor cells, and IFT140^+^/K14^+^ cells were required for epithelial regeneration.

### IFT140 was required for the coordination of ciliary signal trafficking to ensure K14^+^ progenitor cell function

**Fig. 8:**
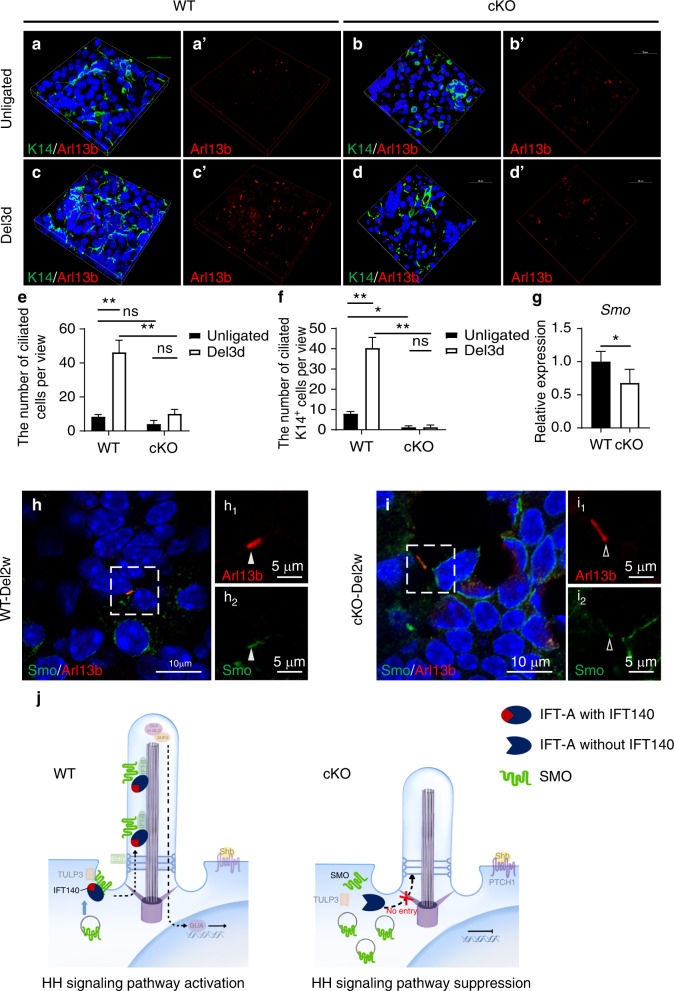
IFT140 was required for coordination of ciliary signal trafficking to ensure K14^+^ progenitor cell function. **a**–**d** Immunolabeling of cilia (Arl13b) and K14^+^ cells in SMGs from unligated or 3-day deligated glands (Del3d) of wild-type and *Ift140-cKO* mice. 3D image was constructed to provide a clear view of cilia. **e**, **f** Quantitative analysis of the number of ciliated cells and ciliated K14^+^ cells. One-way ANOVA, *n* = 6. *P < 0.05, **P < 0.01. **g** The relative mRNA expression of Smo in 2-week deligated glands (Del2w) of wild-type and *Ift140-cKO* mice. Unpaired Student’s *t* test, *n* = 6, *P < 0.05. **h**, **i** Immunolabeling of Smo and cilia (Arl13b) in 2-week deligated glands of wild-type and *Ift140-cKO* mice. The solid arrowhead indicates Smo distributed through the full length of the cilium. The hollow arrowhead indicates Smo blocked at the base of the cilium. **j** The schematic diagram shows the mechanism of Shh signaling pathway suppression because of the failure of ciliary trafficking of Smo in *Ift140* deleted cells. Scale bar: 10 or 5 μm as indicated

Based on previous research that showed IFT140 played a role in stabilizing the IFT-A complex and contributes to signaling transduction through primary cilia,^33^ we evaluated the levels of cellular ciliation in the un-ligated and day 3 post de-ligation glands of WT and *Ift140-cKO* mice, respectively. No difference was observed in the total number of ciliated cells between un-ligated WT and *Ift140-cKO* SMGs, while the number of ciliated K14^+^ cells was lower in un-ligated *Ift140-cKO* glands than that in the un-ligated WT group (Fig. 8a, b, e, f). A robust increase in the number of ciliated cells was observed on day 3 post de-ligation compared with that in the un-ligated ones of the WT mice, and so was the number of ciliated K14^+^ cells (Fig. 8a, c, e, f). However, these increases were suppressed in the *Ift140-cKO* glands (Fig. 8b, d, e, f). In line with their infrequent occurrence in the samples, we found no difference in the number of ciliated K14^+^ cells between samples from un-ligated and day 3 post de-ligation *Ift140-cKO* glands (Fig. 8b, d, f). These results indicated inhibited cilogenesis of K14^+^ stem/progenitor cells in regenerating SMGs due to *Ift140* deletion.

Cell-cell communication between luminal cells and basal stem cells regulates regeneration-induced glandular stem cell multipotency.^34^ Primary cilia dynamically coordinate multiple signaling pathways related to tissue development and regeneration, such as Sonic hedgehog (Shh), Wnt, and others.^14^ Since Smoothened (Smo) is an essential membrane receptor in the Shh pathway that need to be translocated onto the ciliary membrane by the IFT-A complex to activate downstream signaling,^33,35^ we further investigated whether ciliary signaling was blocked due to *Ift140*-deletion by evaluating the expression and subcellular localization of smo in cilia. Real-time PCR relative expression analysis showed that Smo transcription was down-regulated in the *Ift140-cKO* glands 2 weeks post de-ligation (Fig. 8g). Immunostaining demonstrated that Smo staining was accumulated at the base of cilia in *Ift140-cKO* glands, revealing that the ciliary entry of Smo was blocked (Fig. 8h, i, diagrammed in j). These results indicated that IFT140 is needed to coordinate ciliary signal trafficking to ensure K14^+^ progenitor cell function during salivary gland regeneration.

## Discussion

The definition of salivary gland stem cells is evolving, and there remains a challenge of identifying the salivary gland resident stem cells.^36^ K14^+^ cells are considered the bona fide stem/progenitor cells in the salivary gland, but they include a variety of subpopulations, and terminally differentiated MECs are also K14 positive.^3,7^

Our data showed that IFT140 was highly expressed in most K14^+^ cells in the E16 SMGs or at the early stage of gland regeneration, while only a few K14^+^ cells express IFT140 during homeostasis. Meanwhile, K14^+^ MECs did not express IFT140 in normal adult SMGs or in the regenerating glands following a moderate ligation-induced injury. Thus, IFT140^+^/K14^+^ could be a candidate combined marker for salivary gland stem/progenitor cells. Interestingly and unexpectedly, we also detected some IFT140^+^ MECs in a limited area where the gland structure was severely atrophic. Since MECs will be recruited and transdifferentiate into multi-potent progenitor cells under severe stress to ensure rapid regeneration of secretory cells,^7^ here, the IFT140^+^ MECs could be in a progenitor-like state. Indeed, evidence for injury-induced “cellular reprogramming” in multiple tissues has revealed the remarkable flexibility of cell states.^37^ As such, IFT140 could be an important molecule regulating transdifferentiation of MECs.

Kwak’s group identified the K14^+^ cells reside in the ID/GD junction and the basal layer of the secretory ducts as the stem/progenitor cells with high proliferate activity, which can replenish the terminally differentiated K19^+^ cells in GDs during development and homeostasis.^8,9^ Our genetic lineage tracing assay demonstrated that a streaming of IFT140^+^ cells co-expressing K14 could differentiate into GD cells during the postnatal development period, suggesting that IFT140^+^/K14^+^ is a more indicative marker for salivary stem/progenitor cells.

Although there were multiple structural and functional abnormalities observed during the development and regeneration of *Ift140-cKO* SMGs, the deficiency was comparatively mild. This could be due to contributions of other salivary stem/progenitor cell lines, such as K5^+^ or Axin2^+^ cell lines.^11,38^ In addition, our results indicated that the expression of AQP5 and the area of acini were down-regulated in the 1-week-old *Ift140-cKO* mice, but no difference was detected between the WT and *Ift140-cKO* adult mice, neither did the saliva flow rate or the constituents of saliva. Thus, *Ift140*-deletion did not affect the fluid secretory function in adults but just delayed the development process of acini. *Ift140*-deletion from K14^+^ cells seemed to have more impacts on GD function since the expression of NGF and claudin-3 were altered, and the secretory granules were accumulated in GD cells in *Ift140-cKO* mice.

Primary cilia are surface-exposed organelles that dynamically concentrate signaling molecules to organize developmental and homeostatic pathways, and the IFT-A complex promotes the entry of signaling receptors into cilia.^15,39,40^ IFT140 is a core component of the IFT-A complex, and its mutation destabilizes IFT-A, suppressing the formation of cilia.^33^ It is related to various ciliopathies, including Jeune asphyxiating thoracic dystrophy, Mainzer-Saldino syndrome, Sensenbrenner syndrome, and spermatogenic dysfunction.^20,22,23,26^ The Shh pathway is a well-known ciliary signaling system that regulates cell fate and self-renewal in development and tissue homeostasis.^14,41^ When Shh ligand is present, it binds to the membrane receptor, Ptch1, and permits Smo to translocate onto the ciliary membrane, leading to the processing of transcription factors downstream the pathway.^35,42^ In this process, IFT140 plays an essential role in the ciliary entry of Smo.^13,43^ Our results showed that the number of ciliated K14^+^ cells failed to be increased at the onset of regeneration in *Ift140-cKO* SMGs. Moreover, Smo was blocked at the base of the remaining cilia, which supported the assumption that *Ift140* deletion unstablized the IFT-A complex and then failed to coordinate the ciliary signaling trafficking in Shh pathway. Here, the abnormal localization of Smo is a key issue in the deficiency of gland regeneration in *Ift140-cKO* mice, even though the gene expression of Smo was just moderately down-regulated. These results suggest that IFT140 is required to coordinate ciliary signal trafficking to ensure K14^+^ progenitor cell function. It is worth noting that the present study could not rule out the non-ciliary function of IFT140, which could also regulate the function of K14^+^ stem/progenitor cells, since many ciliary proteins have been found at extraciliary sites in cells and have non-ciliary functions.^44^

In conclusion, we recommend IFT140^+^/K14^+^ as a potential marker for salivary stem/progenitor cells. Moreover, we demonstrated the crucial role of the core ciliary molecule IFT140 in promoting salivary gland development and regeneration by regulating the function of K14^+^ stem/progenitor cells. This study could provide a new perspective on gland-specific stem cell subset and stem cell therapy targets for salivary gland damage.

## Materials and methods

### Animals

The mice used in this study were maintained on a C57/Bl6 background. *Ift140*^*flox/fox*^ mice were produced as previously described.^17^ The primers for genotyping included: Forward, 5′-ATCTTAATTTGTGTTGAAGGGGTT-3′, and Reverse, 5′-CTGCCAGGGGTACATGGTAGTAAG-3′. They were crossed with *K14-Cre* mice (purchased from the Jackson Laboratory) to generate K14^+^ SSPC-selective *Ift140-cKO* mice. The genotyping method of the *Ift140*^*flox/fox*^ and *Ift140-cKO* mice were presented in Appendix Fig. S1. For the genetic linage trancing study, *Ift140-Cre*^*ERT2*^ mice were generated as previously reported.^16^ The identification of *Ift140-Cre*^*ERT2*^ mice included the forward primer, 5′-CTAATGGTTCCAGTCTGCAGGCCC-3′, and the reverse primer, 5′-CAATAAGACCAGGCACATACCATC-3′. Then, *Ift140-Cre*^*ERT2*^ mice were crossed with the reporter strain *Rosa26*^*tdTomato*^ (purchased from Cyagen Biotechnology). Mice were raised in a specific pathogen-free facility under a 12:12-h day/night illumination cycle. All procedures and protocols were approved by The Animal Welfare Committee of Tongji University, Hospital of Stomatology (2019-DW-004).

### Genetic linage tracing

Nine *Ift140-cre*^*ERT2*^*; Rosa26*^*tdTomato*^ mice were induced by TAM injection. The cells that express IFT140 and their progeny emitted red fluorescence following induction. The proliferation and differentiation of the IFT140^+^ cells could be tracked. TAM was dissolved in 10% ethyl alcohol and 90% olive oil at a concentration of 1 mg·mL^−1^. Each mouse received an intraperitoneal injection at a dose of 50 μg per day on P3, P5, and P7, respectively, and SMGs were harvested at P9, P14, and P21 (6 glands for each time point). Samples were processed into frozen sections and counterstained with cell-specific biomarkers to map the fate of IFT140^+^ cells during the development period.

### Ligation and de-ligation of the main excretory ducts of SMGs

Adult female WT and *Ift140-cKO mice* (8 weeks, *n* = 6) were subjected to fasting for 12 h and anesthetized with an intraperitoneal injection of chloral hydrate (0.4 g·kg^−^^1^ body weight) and local infiltration with lidocaine. The main excretory ducts of SMGs were separated through the cervical approach and ligated by the micro titanium clips using previously published methods.^11^ The clips were maintained for 2 weeks and removed with care to permit gland regeneration. The SMGs were dissected at different time points, as indicated in the results. The gross feature of the glands was observed to confirm that the gland injury model was successfully induced, and the samples were prepared for further study.

### Saliva flow rate and composition measurement

The saliva of adult male and female *Ift140-cKO* mice as well as adult WT mice were collected and measured (8 weeks, *n* = 6). After stimulation with the cholinergic agonist pilocarpine (10 μg·g^−1^, body weight, intraperitoneal), the whole saliva secretion within 15 min was collected into a centrifuge tube. The saliva flow rate was calculated as the volume of total collected saliva (μL)/collection time (min). The ion concentration was measured by an automatic biochemical analyzer (Roche, Cobase c311).

### Real-time quantitative polymerase chain reaction

The gland tissues were homogenized in TRIzol reagent (Sigma). First-stand complementary DNA (cDNA) was synthesized from 1 μg total RNA in a volume of 20 μL with Oligo DT primers using a Transcriptor First Strand cDNA Synthesis Kit (Roche). Primers are listed in Appendix Table S1. The mouse Gapdh gene was used as an endogenous control for each sample.

### Histological staining, immunofluorescence staining, and TUNEL assay

Tissues were fixed and embedded in paraffin. 5-μm-thick sections were subjected to hematoxylin and eosin (H&E) and AB/PAS staining. In total, 10-μm-thick sections were subjected to immunofluorescence staining. Sections were blocked in bovine serum albumin for 1 h and incubated with primary antibodies (Appendix Table S2) at 4 °C overnight. Secondary antibodies were bound to the primary antibodies for 1 h at room temperature. DAPI was used to stain the nuclei. For apoptosis, TUNEL staining was performed with the TUNEL Apoptosis Detection Kit. ImageJ software was used to quantify the area of the secretory granules by gray-scale value on the AB/PAS staining images, and the acini were outlined to calculate the acinar area. The expression level of AQP5 or NGF was quantified by fluorescence intensity on the immunofluorescence images. The number of K14^+^ cells, IFT140^+^/K14^+^ cells, ciliated K14^+^ cells, and the apoptotic cells in each view were counted as indicated in the results.

### Statistical analysis

The data were presented as means ± standard deviation. All data were performed by GraphPad Prism 8.0. Kolmogorov–Smirnov test was used to evaluate the normality distribution. For data that were normally distributed, comparison between two groups was performed by Student’s *t* test if the variance was equal, and if not, Welch’s *t*-test was used. For non-normal distribution data, Mann–Whitney test was used. Differences for multiple groups were compared using one-way ANOVA followed by the Bonferroni test. *P* < 0.05 was considered statistically significant.

## Supplementary information

Appendix File
