# Supplementary material for: IFT140+/K14+ cells function as stem/progenitor cells in salivary glands
Source: Int J Oral Sci. 2022 Oct 10;14:49. doi: 10.1038/s41368-022-00200-5 (PMC9550827; doi:10.1038/s41368-022-00200-5)
Supplement: Supplementary file 1 — Appendix File [file 41368_2022_200_MOESM1_ESM.docx]

**IFT140^+^/K14^+^ cells function as stem/progenitor cells in salivary glands**

**Appendix File**

**Tables**

Table S1. The primers used in RT-PCR assay.

| Gene | Forward primer (5’-3’) | Reverse primer (3’-5’) |
| --- | --- | --- |
| *Ift140* | AACACCACTCCTGAAACACGA | ATGTCCAGGGCTTTCTCATCC |
| *Ift144* | CAGTATTCACGGGCACTAAAGC | GTACTTGGCGTCCTTTGGC |
| *Ift22* | TTAACGGACAGCGATGCTGAA | GGGCCAACAGGACTCAAACT |
| *Ift88* | TGAGGACGACCTTTACTCTGG | GAAAACCCGTGTCATTCTCCAA |
| *Ift74* | GCCAGCATGGAGAGACAGTT | AGACACAAAATTCCATAAGCTGAGA |
| *Smo* | GGGCTGGGAGTCGGTTTTAAT | CTTCAACCCTGGGAACCCTC |

Table S2. The message of the antibodies used in this study.

| Antibodies | Dilution | Catalog | Source |
| --- | --- | --- | --- |
| Anti-aquaporin 5 (AQP5) | 1:200 | 3069691 | Merck KGaA |
| Anti-Arl13b | 1:300 | 17711-1-AP | Proteintech Group |
| Anti-alpha-SMA | 1:300 | A5228 | Sigma |
| Anti-cKit | 1:100 | 14-1171-82 | Invitrogen |
| Anti-cytokeratin14 (K14) | 1:1000 | MA5-11599 | Thermo Fisher Scientific |
| Anti-cytokeratin19 (K19) | 1:500 | bsm-33057M | Bioss |
| Anti-IFT140 | 1:300 | 17460-1-AP | Proteintech Group |
| Anti-Smoothened (Smo) | 1:50 | sc-166685 | Santa Cruz Biotechnology |

**Figures**


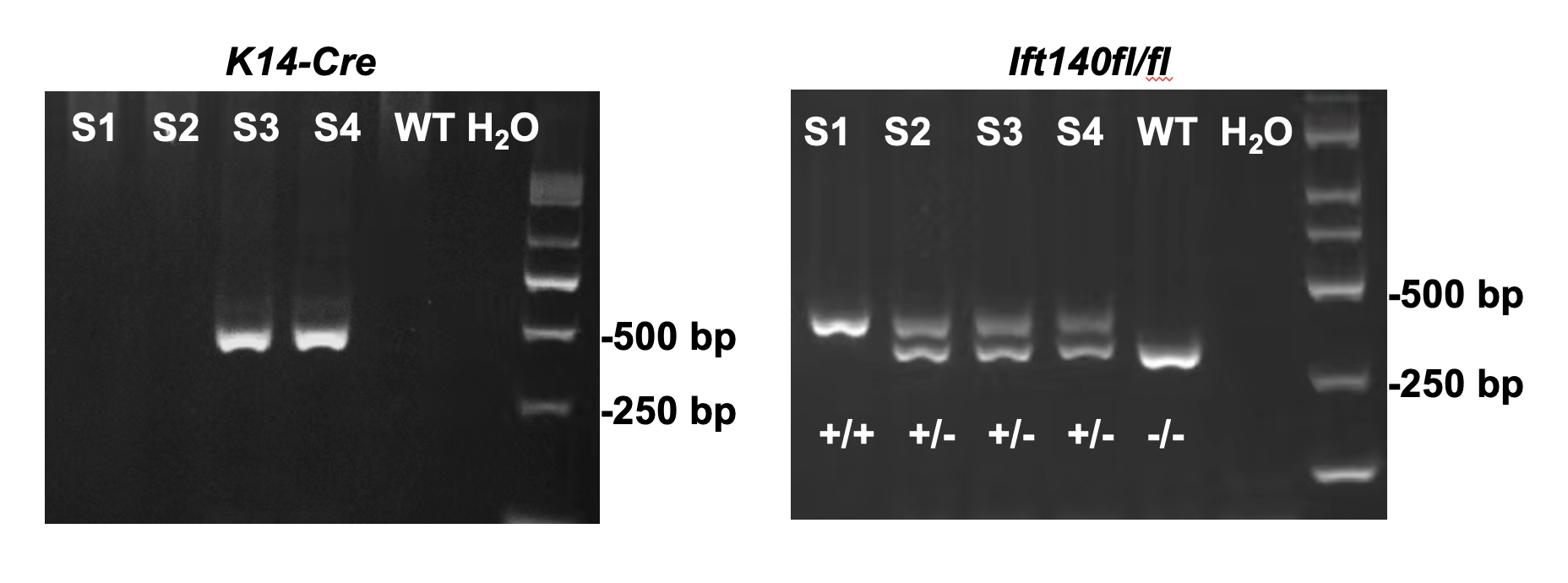


**Figure S1** The genotyping method of K14-Cre; Ift140^flox/flox^ (Ift140-cKO) mice is shown. The representative pictures of genotyping of sample 1-4 by PCR are presented. Only samples containing both K14-Cre positive band and IFT140^fl/fl^ band should be considered from Ift140-cKO mice.
